# Mycobacterium abscessus Infection in Left Ventricular Assist Device (LVAD): A Case Series

**DOI:** 10.7759/cureus.15718

**Published:** 2021-06-17

**Authors:** Jarelys M Hernandez, Mallorie Huff, Zola Nlandu, Bhavesh Vekariya, Jeniffer Torres

**Affiliations:** 1 Infectious Diseases, University of South Florida Morsani College of Medicine, Tampa, USA; 2 Infectious Diseases, Orlando Regional Medical Center, Orlando, USA

**Keywords:** left ventricular assist device, mycobacterium abscessus, driveline infection, rapidly growing mycobacteria, nontuberculous mycobacteria

## Abstract

The limited availability of donor organs worldwide, has provoked a surge in the need for implantable left ventricular assist devices (LVADs) mainly as a bridge to heart transplantation or destination therapy. The rate of complications from LVAD use is also increasing, impacting morbidity, mortality, and costs. LVAD infections due to nontuberculous mycobacteria (NTM) are exceedingly rare, yet very difficult to treat. Here we present three cases of *Mycobacterium abscessus* LVAD infections. To our knowledge, only two other cases have been documented.

## Introduction

Due to increasing prevalence of heart failure and limited availability of donor organs, implantable left ventricular assist devices (LVADs) are increasingly used as a bridge to heart transplantation, destination therapy, and bridge to recovery [1,2]. Concurrently, the rate of complications from LVAD use is increasing, with LVAD infections becoming a major problem with worsening mortality and increased costs [3].

Most LVAD infections are caused by *Staphylococcal* species, specifically *Staphylococcus aureus* and Gram-negative bacteria, including *Pseudomonas* [2-4]. Driveline infections, the most common infections in patients with LVADs, occur in 9-48% of patients within 6-8 months of implantation and are associated with increased risk of death [2,5].

Nontuberculous mycobacteria (NTM) are ubiquitous environmental organisms found in dust, soil and water [5]. NTM infections typically manifest as pulmonary disease in patients with underlying chronic lung disease, while LVAD infections are exceedingly rare [1,3,5]. Rapidly growing mycobacteria (RGM) are a group of NTM species that include *M. abscessus *complex, *M. chelonae*, and *M. fortuitum* [6]. *M. abscessus* complex is further subclassified into three closely related subspecies: *M. abscessus* subs. *abscessus*, *M. abscessus* subs. *massiliense* and *M. abscessus* subs. *bolletti* [6,7]. Importantly, *M. abscessus* complex organisms are resistant to first-line anti-tuberculous agents and most other antibacterial agents [6,7]. Hence, infections associated with *M. abscessus* complex are difficult to treat and use of multiple antimicrobial agents for an extended duration is often required [7,8]. Moreover, these antimicrobial agents tend to not be well tolerated by patients.

## Case presentation

Case 1

An 80-year-old Caucasian male with chronic kidney disease and dilated cardiomyopathy with LVAD as destination therapy presented to the emergency room with nausea and chills. His LVAD had been placed eight years prior to presentation and his clinical course was complicated by chronic driveline infections predominantly due to *Serratia* and *Staphylococcus epidermidis*, and the use of chronic antimicrobial prophylaxis with cefdinir.

**Figure 1:**
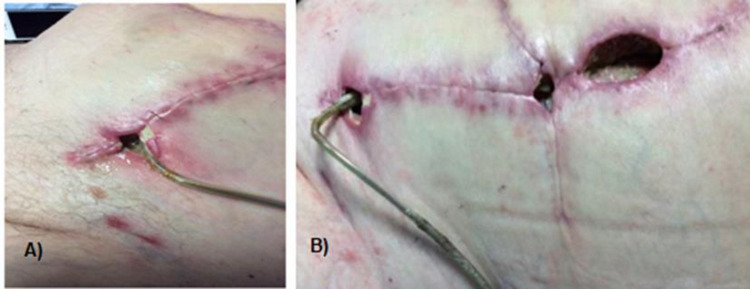
Open wound adjacent to the driveline site in the left lower quadrant (A), with progression to severe wound dehiscence after flap and driveline relocation to right lower quadrant (B).

On admission, he was afebrile and hemodynamically stable. On exam, his driveline dressing was soaked with yellow fluid which was also emanating from his left lower quadrant suggesting the presence of a sinus tract (see Figure 1). His white blood cell count was 10.04 x 10^3^/μL, hemoglobin 10.5 g/dL, and creatinine 0.8 mg/dL. Blood cultures were unremarkable. A computed tomography (CT) scan of his abdomen and pelvis did not demonstrate any collections involving his LVAD. While in hospital, our team was made aware of positive acid-fast bacilli (AFB) cultures from his driveline done a month ago at an outside facility. Therefore, the patient was taken to the operating room for irrigation and debridement (I&D), and flap closure of his LVAD site. He was deemed a high-risk surgical candidate for device removal. Accordingly, he was empirically treated with imipenem and tigecycline intravenously, and oral azithromycin. The AFB cultures and *Mycobacterium* PCR collected intraoperatively yielded positive results for *Mycobacterium abscessus* subs. *massiliense* (see Table 1).

**Table 1:** Initial minimum inhibitory concentrations (MIC) of antibiotics tested against M. abscessus isolates from cases 1, 2, and 3 S = Susceptible, I = Intermediate, R = Resistant, NI = No CLSI interpretive guidance for this antibiotic, TS = Tentative Interpretation Susceptible, TI = Tentative Interpretation Intermediate, TR = Tentative Interpretation Resistant

Antibiotic	MIC (mcg/mL) for Case 1	MIC (mcg/mL) for Case 2	MIC (mcg/mL) for Case 3
Amikacin	16 S	16 S	32 I
Augmentin	>32/16 NI	>32/16 TR	>32/16 NI
Azithromycin	≤16 NI	64 TI	≤16 NI
Cefepime	>32 NI	>32 TR	>32 NI
Cefotaxime	>64 NI	>64 TR	>64 NI
Cefoxitin	<16 S	≤16 S	64 I
Ceftriaxone	>64 NI	>64 TR	>64 NI
Ciprofloxacin	8 R	4 R	>8 R
Clarithromycin	≤0.25 S	32 R	0.5 S
Clofazamine	≤0.5 NI	≤0.5 TS	1 NI
Clofazamine/Amikacin	N/A	≤0.5/2	≤0.5/2 NI
Doxycycline	>16 R	>16 R	>16 R
Gentamicin	>16 NI	16 TR	>16 NI
Imipenem	8 I	8 I	16 I
Kanamycin	≤8 NI	≤8 TS	16 NI
Linezolid	>16 R	>16 TR	>16 NI
Minocycline	>8 NI	>8 TR	>8 NI
Moxifloxacin	>4 R	>4 R	>4 R
Tigecycline	2 NI	2 TS	1 NI
Tobramycin	>16 R	8 R	16 R
Trimethoprim/Sulfamethoxazole	>4/76 R	>4/76 R	>4/76 R

Due to gastrointestinal-related intolerance, the patient preferred to remain off antibiotics intermittently during his inpatient stay. Eventually, tigecycline was switched to amikacin. However, after a few days his gastrointestinal complaints recurred. His most recent regimen was amikacin, cefoxitin, and azithromycin. Ultimately, the patient opted for home with hospice, antibiotics were discontinued, and he expired.

Case 2

**Figure 2:**
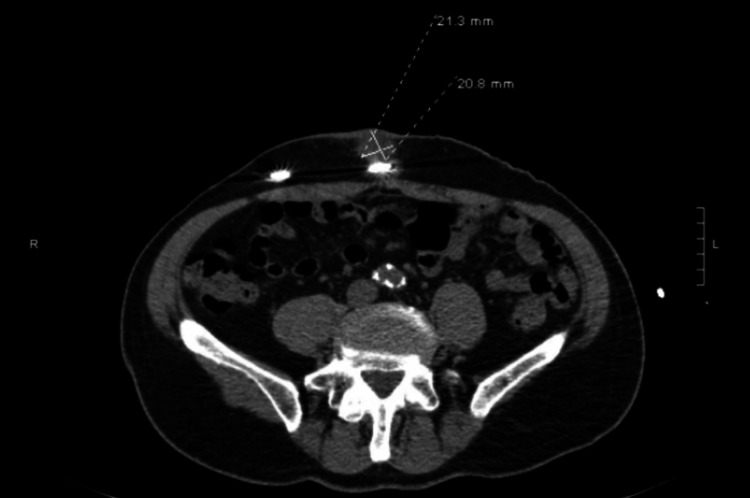
CT of abdomen and pelvis without contrast demonstrating a 2.1 x 2.0 cm sized fluid collection along the subcutaneous aspect of the drive line in the infra-umbilical region.

An 80-year-old Caucasian male with bioprosthetic aortic and mitral valve replacement, and ischemic dilated cardiomyopathy status post LVAD placement for destination therapy four years prior presented with increasing yellowish drainage from LVAD exit site. One month prior he had been treated for a similar complaint with a three-week course of doxycycline followed by linezolid, after driveline site cultures yielded *Staphylococcus epidermidis*, but he showed minimal improvement with treatment. On presentation, he was afebrile and hemodynamically stable. His white blood cell count was 3.98 x 10^3^/μL, hemoglobin 11.2 g/d, and creatinine 1.3 mg/dL. A CT scan of his abdomen and pelvis revealed a 2 x 2 cm collection along the subcutaneous aspect of the driveline (see Figure 2).

Two days following admission, an excisional debridement was done and cultures yielded *M. abscessus* subs. *abscessus*. The patient was started on empiric imipenem and tigecycline intravenously, and oral azithromycin. One week later, he had further I&D, followed by addition of amikacin. Once susceptibilities were available (see Table 1) imipenem was changed to cefoxitin. Subsequently, his regimen was changed to clofazimine, cefoxitin, tigecycline, and azithromycin after Food and Drugs Administration (FDA) approval was acquired for clofazamine. Unfortunately, for two years after discharge he had multiple readmissions almost always related to *M. abscessus* subs. *abscessus* driveline infections, as well as secondary infection with *Serratia* requiring further debridement. Unable to tolerate tigecycline due to intractable nausea and worsening anorexia, the agent was discontinued. The patient is currently on lifelong suppressive therapy with clofazamine and doxycycline.

Case 3

**Figure 3:**
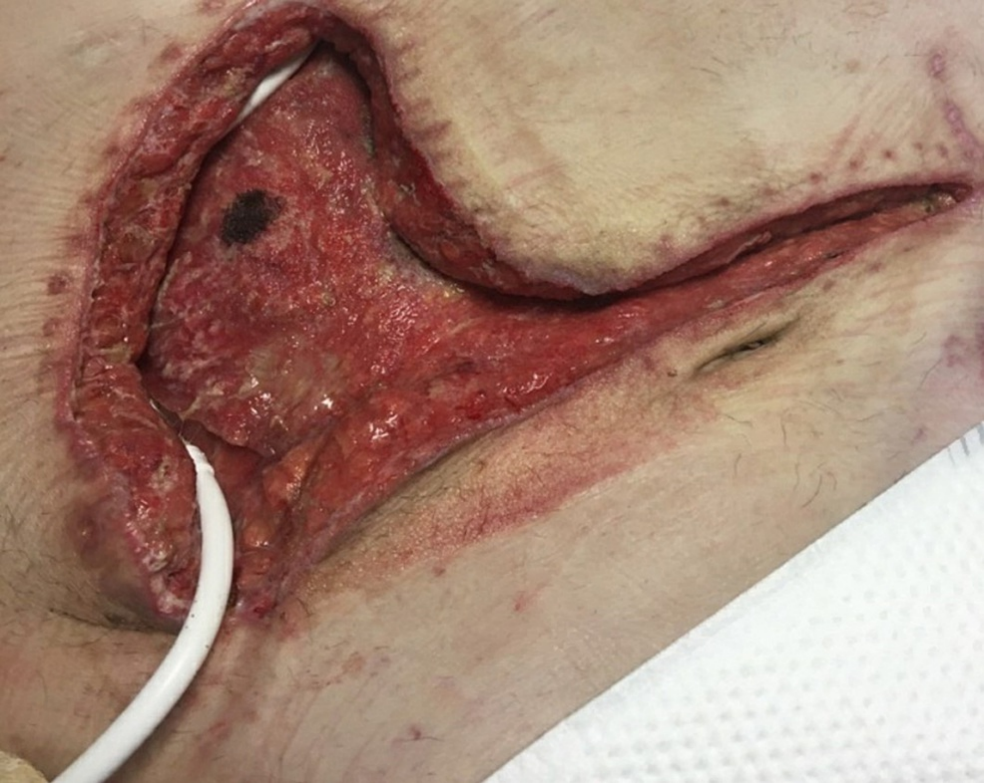
Extensive, open driveline-associated wound.

A 51-year-old Caucasian male with hypertrophic cardiomyopathy and recurrent ventricular tachycardia with LVAD placement two years prior presentation for bridge to transplantation presented with complaints of low grade fevers with concern for recurrent infection involving the LVAD line exit site (see Figure 3). More than three weeks prior, he had debridement of the site and cultures grew *Staphylococcus pseudintermedius* and *Staphylococcus epidermidis*. He underwent debridement, line repositioning and flap closure. On presentation, he was afebrile and hemodynamically stable. On exam, serosanguinous drainage was noted on the LVAD site dressing overlying the abdomen. His white blood cell count was 15.72 x 10^3^/μL, hemoglobin 13.2 g/dL, and creatinine 1.7 mg/dL. A CT scan of the abdomen and pelvis denoted an ill-defined high-density fluid measuring 4.4 x 5.7 cm with subtle adjacent fat stranding in the right upper and mid aspect of the abdomen, which crossed adjacent to the driveline. Thus, additional cultures were obtained with further debridement.

AFB cultures from the LVAD exit site were notable for *Mycobacterium abscessus* subs. *massiliense*, and he was started empirically on imipenem and tigecycline intravenously, and oral azithromycin. The patient was discharged, but was subsequently readmitted due to gastrointestinal intolerance to tigecycline. As such, this was replaced by omadacycline, which he later was unable to tolerate as well. Over the next five months, the patient’s clinical status deteriorated and he had numerous readmissions. A decision was made for him to be on lifelong therapy with oral clofazamine and azithromycin (see Table 1). However, his last admission to the hospital was for confusion and electrolyte disturbances for which he required urgent dialysis. His poor inpatient clinical course led to comfort care measures being instituted as per request, and he subsequently expired.

## Discussion

NTM infections require a high index of clinical suspicion and diagnostic pathway [1]. In patients with LVAD driveline infections unresponsive to conventional antibiotic regimens and negative bacterial cultures on non-selective culture media, NTM should strongly be considered [5]. As presented herein, treatment requires a combination of antibiotics based on susceptibility results, for at least four months in the case of skin and soft tissue infections [5]. However, the recommended duration of antimicrobial therapy specifically for LVAD-related infections due to NTM is unknown.

Moreover, medical therapy may not be enough [4]. NTM have the tendency to form biofilms, and removing the foreign or prosthetic material seems prudent whenever feasible, with driveline relocation to the opposite side [5], as was also seen in our cases. However, the efficacy of device exchange as an adjunct to medical therapy for invasive NTM is unknown [9].

Interestingly, tissue hypoxia like seen in this patient population has shown to play an important role in both innate and adaptive host immunity through the regulation of transcription factors in both infiltrating immunocytes and inflamed resident cells, which can result in bacterial infection and subsequent progression [10]. In addition, data supports that malnutrition frequently co-occurs with chronic heart failure, and malnutrition in turn has been associated with impaired wound healing, and increased rate of postoperative morbidity and mortality [11].

Subspecies level identification of NTM is essential as antibiotic susceptibility and outcomes of therapy differ significantly depending on the organism isolated [6]. For instance, *M. abscessu*s subs. *massiliense* has a much better prognosis using macrolide multidrug regimens because of the absence of inducible *erm* gene, which confers macrolide resistance [3,6,8]. The diagnosis of the cases presented was made via matrix-assisted laser desorption ionization-time of flight mass spectrometry (MALDI-TOF) at our intuition. Subspecies identification and susceptibility testing was performed at National Jewish Health, in Denver, CO.

For *M. abscessus* complex, a combination therapy with an oral macrolide, parenteral amikacin, and imipenem or cefoxitin according to in vitro drug susceptibility testing results is recommended [6-8]. In addition, a high susceptibility to linezolid, tigecycline and clofazimine has been observed for this entity [7]. Of note, clofazimine is very effective against *M. abscessus* including resistant strains, but is not available in US pharmacies and can only be obtained by contacting the FDA and requesting a single patient investigational new drug (IND) [3].

*Mycobacterium abscessus* infections are notoriously multidrug resistant, and necessitate prolonged intravenous therapy. Additionally, side effects are common [8]. Furthermore, there are no drug combinations with proven efficacy, and treatment regimens have no clear pattern given the multiple medication changes as a result of intolerable side effects [7,8], as seen with our patients. Unsurprisingly, the long-term treatment response rate for *Mycobacterium abscessus* infections is unsatisfactory [7].

NTM infections can be acquired from the community or nosocomially [5,9]. They have been isolated from drinking water and showerheads [12,13]. In the United States, NTM infections have a coastal predominance as they are ubiquitous in coastal swamps and estuaries [14], a prominent ecological feature of south Florida. Furthermore, data suggests that hospitals labeled as “urban”, “larger”, and “teaching” (like our institution) more often report NTM cases [15]. In addition, LVAD procedures are only offered in a selected group of hospitals. All these factors account for the rarity of this diagnosis, and how we encountered three independent cases at our institution.

Only two other cases of *M. abscessus* LVAD infections were found in the literature, described by Nunez et al. [3]. These patients received antibiotics for more than one year. For patient 1, the *M. abscessus* subspecies was not provided, and he received cefoxitin, tigecycline, and azithromycin. Adjuvant surgical management was via driveline unroofing. He did receive a heart transplant, but died from acute rejection. Patient 2 received cefoxitin, amikacin, and clarithromycin. Surgical management was with pocket debridement and device exchange. He expired from invasive aspergillosis and stroke. Our three cases of *M. abscessus* underwent surgical debridement and similar antibiotic therapy. Cases 1 and 3 underwent flap and line relocation. Of note, patients in our institution are instructed to use chlorhexidine gluconate soap and sterile water daily or twice a day when caring for the LVAD exit site.

Without a correct diagnosis and treatment, the patients’ quality of life and life expectancy can be negatively impacted [1,4,9], especially in the context of an already challenging to treat organism. Unfortunately, two of our patients expired (patient 1 and 3), and only Patient 3 had his LVAD placed as a bridge to transplant, but he was excluded from the transplant list after his NTM infection diagnosis. Patient 2 is undergoing palliative antimicrobial therapy in conjunction to wound care. Larger case series and better tolerated antimicrobials are needed.

## Conclusions

The rate of complications from LVAD use is increasing with LVAD infections becoming a major problem with worsening mortality and increased costs. LVAD infections due to NTM are extremely rare and require a high index of clinical suspicion and diagnostic pathway. It is of utmost importance to identify the subspecies level, as antibiotic susceptibility and outcomes of therapy vary significantly depending on the isolated organism, and the presence of inducible* erm* gene. Without a correct diagnosis and timely treatment, patients with NTM driveline infections could be excluded from the transplant list, resulting in grave repercussion to their morbidity, quality of life, and mortality.

It is of interest to learn whether any centers have had a successful experience on transplanting patients with NTM-related LVAD infections given the concern for post-surgical complications and dissemination with immunosuppression. *M. abscessus* infections are more challenging to treat than other NTM not only because of the limited antimicrobial options, but also tolerability issues which can be further aggravated in the post-transplant setting. Overall, larger case series and more tolerable antimicrobials are needed, as a matter of life and death.
